# Spleen Metabolome Reveals Immune-Mediated Responses Modulated by Onion Peel Extract in *Salmonella*-Infected Broiler Chicks

**DOI:** 10.3390/microorganisms14071397

**Published:** 2026-06-24

**Authors:** Odinaka C. Iwuozo, Paul C. Omaliko, Oluteru E. Orimaye, Safiu A. Suberu, Hye Won Kang, Yewande O. Fasina

**Affiliations:** 1 Department of Animal Sciences, North Carolina Agricultural and Technical State University, Greensboro, NC 27411, USA; ociwuozo@aggies.ncat.edu (O.C.I.); pcomaliko@aggies.ncat.edu (P.C.O.); oeorimaye@aggies.ncat.edu (O.E.O.); sasuberu@aggies.ncat.edu (S.A.S.); 2 Food and Nutritional Sciences, Department of Family and Consumer Sciences, North Carolina Agricultural and Technical State University, Greensboro, NC 27411, USA; hkang@ncat.edu

**Keywords:** spleen metabolome, dietary onion peel extract, *Salmonella* Enteritidis, immune response, broiler chicks, polyphenol

## Abstract

Onion peel extract (OPE) is rich in polyphenolic compounds with antimicrobial potential. *Salmonella* Enteritidis (SE) infection in young broiler chicks causes morbidity, reduced growth, and contributes to human gastroenteritis through contaminated poultry products. The spleen is a key secondary lymphoid organ coordinating systemic responses to pathogens in chicken. This study evaluated how dietary OPE influences spleen metabolic profiles during SE infection. Day-old Ross 708 male chicks (n = 128) were assigned to four treatments: CON, CON-SE, OPE (6 g/kg), and OPE-SE. Chicks in CON and OPE received sterile broth, whereas CON-SE and OPE-SE received 2.25 × 10^8^ CFU/mL SE at 2 d of age. At 5 and 12 dpi, spleens from six chicks per treatment were collected for untargeted HPLC-MS metabolomics. A total of 857 metabolites were identified and analyzed using MetaboAnalyst 6.0 (*p* < 0.05; fold change ≥ 2.0; VIP score > 1.0). In CON-SE chicks, energy generating metabolites (6-phosphogluconic acid, methylmalonic acid, propionic acid) increased, while 13,14-dihydro-15-keto-prostaglandin D2 and kynurenic acid decreased. Dietary OPE elevated several dipeptides (L-Val-Gly, L-Leu-Gly, Gly-Gly-Leu, L-Val-L-Met) and reduced ATP linked metabolites (3,6-di-O-methyl-beta-D-glucose and 3-O-beta-D-galactosyl-sn-glycerol). Enrichment analysis showed that SE infection altered valine, leucine, and isoleucine degradation and aromatic amino acid biosynthesis, whereas OPE enriched galactose and biotin metabolism in uninfected chicks, but enriched tryptophan, taurine and hypotaurine metabolism in SE-infected chicks. Overall, dietary OPE optimized response of metabolic pathways associated with immune activation, unlike corresponding pathways in CON-SE birds.

## 1. Introduction

*Salmonella enterica* serovar Enteritidis (SE) infection remains a significant challenge in poultry production, leading to substantial economic losses and posing serious public health risks to both animal health and food safety in humans due to its zoonotic transmission potential [1]. In broiler chicks, SE infection can lead to severe enteritis, systemic inflammation, and compromised immune function that can impair growth performance and overall health, resulting in economic losses for the poultry industry [2]. As concerns about antibiotic resistance and residues in food products continue to rise, there is an increasing demand for alternative strategies to mitigate infection, reduce foodborne pathogens, and enhance disease resistance and immune function in poultry [3]. Among the alternatives, natural dietary supplements, particularly plant-derived compounds, have gained attention for their immunomodulatory and antimicrobial properties in chickens [4,5,6]. Natural bioactive compounds, specifically plant-based sources enriched with polyphenols such as flavonoids, have gained considerable attention for their immunomodulatory and antimicrobial properties in broiler chickens [7,8,9].

Accordingly, onion peel extract (OPE), a by-product of onion processing, is particularly rich in polyphenols, most notably quercetin and its glycosides, along with other flavonoids and phenolic acids, which exhibit antioxidants, anti-inflammatory, and antimicrobial activities [10,11,12]. Recent studies have demonstrated that plant-derived bioactive may influence metabolic pathways linked to oxidative stress, lipid metabolism, and immune cell activation [13,14], while also enhancing growth performance, improve gut health, and modulate immune responses in poultry [15,16]. Moreover, the phytochemicals in onion are known for their ability to modulate immune responses and reduce oxidative stress [17], factors that are critical during *Salmonella* infection, suggesting that OPE [4,5,6] may alter the metabolic profile of the spleen, an essential immune organ, thereby enhancing chicken defense mechanisms through antimicrobials and immunomodulatory activity. The spleen plays a crucial role in the immune system by filtering blood, removing damaged red blood cells and bloodborne pathogens, and serving as a site for immune surveillance [18]. As a key lymphoid organ, the spleen plays a central role in orchestrating immune responses to systemic infections, including immune cell proliferation and cytokine secretion, thereby serving as an important site for pathogen recognition [19]. Accordingly, the spleen is a suitable target for metabolomic analysis because it is the largest peripheral lymphoid organ in chickens and a major site of systemic immune responses during pathogen infection. Since *Salmonella* can disseminate beyond the intestine to internal organs, splenic metabolites can provide valuable insight into chickens’ defense, inflammation, and the overall metabolic responses associated with infection and recovery. Elucidating how dietary interventions influence splenic immune mechanisms may offer valuable insight into host–pathogen interactions and the development of immune enhancing strategies in poultry.

Metabolomic profiling of the spleen offers a valuable approach for elucidating how dietary interventions modulate systemic immune responses, particularly during *Salmonella* infection, by revealing key metabolites and biochemical pathways involved in host defense, inflammation, and immune responses [20]. However, the mechanisms by which OPE influences the immune response to SE infection, particularly at the molecular and metabolic levels, remain largely unknown. There is limited knowledge on how the polyphenols and quercetin-rich onion peel modulate the spleen metabolome to mitigate SE-induced metabolic dysregulation. By employing an untargeted metabolomics approach, this study aims to reveal novel insights into the metabolic signatures associated with enhanced disease resistance and immune response mechanisms by OPE in *Salmonella*-infected broiler chicks. Ultimately, these findings could support the application of OPE as a natural alternative to enhance disease resistance while reducing reliance on conventional antimicrobials in poultry production.

## 2. Materials and Methods

### 2.1. Animals, Treatments, and Diet Composition

**Figure 1:**
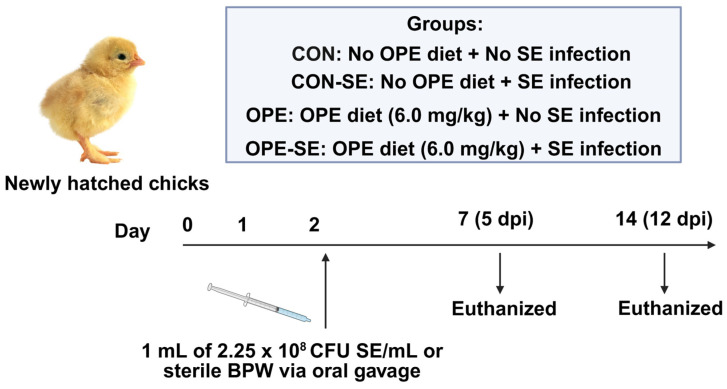
Experimental design. Newly hatched chickens were randomly divided into four groups (CON, CON-SE, OPE and OPE-SE). The chickens were infected with 2.25 × 10^8^ CFU *S.* Enteritidis nalidixic acid-resistant strain by oral gavage.

Day-old (Ross 708; n = 128) male broiler chicks were commercially obtained and housed in the isolator battery cage (Alternative Design Manufacturing and amp Supply Inc., Siloam Springs, AR, USA) at the Poultry Research Unit of the North Carolina A&T State University (Greensboro, NC, USA). Each isolator battery cage pen was fitted with a nipple drinker to supply water and a feeder tray which was adjusted in height for reach according to the progressive growth of the chicks. The bird housing was set at a temperature of 92 °F from d 1 to d 7, and 87 °F from d 8 to d 14. Meanwhile, the photoperiod consisted of continuous (23L:1D) lighting at 30 lux from placement to 14 d. To confirm that the chicks were free of the nalidixic acid (NA; Fisher Scientific, Fair Lawn, NJ, USA)-resistant SE marker strain that was used in this challenge trial, 20 chicks were randomly taken upon arrival from hatchery, then euthanized by CO_2_ asphyxiation, and aseptically necropsied for the removal of ceca. The chicks for the study were weighed and randomly assigned to 4 treatments with 4 replicate pens housing 8 chicks each. The treatment CON consisted of chicks fed unmedicated corn-soybean meal (SBM) basal diet (as shown in the experimental diet, Table 1) without OPE inclusion or SE infection, CON-SE consisted of chicks fed unmedicated corn-SBM basal diet with SE infection only, OPE consisted of chicks fed unmedicated corn-SBM basal diet with OPE inclusion at 6.0 g/kg, but without SE infection and, OPE-SE consisted of chicks fed unmedicated corn-SBM basal diet with OPE inclusion at 6.0 g/kg diet + SE infection. The 6.0 g of OPE/kg diet inclusion level used in this study was selected based on findings from a preliminary study conducted in our laboratory and presented at the poultry science conference, where this dose demonstrated promising antimicrobial effects in broiler chickens. Chicks in CON-SE and OPE-SE groups were each inoculated by oral gavage with 1 mL of NA-resistant SE marker strain inoculum containing 2.25 × 10^8^ colony forming unit (CFU) SE/mL supplemented with 50 µg/mL of NA (0.1%) as described by Fasina et al. [21], whereas CON and OPE treatments were administered 1 mL of sterile buffer peptone water (Thermo Scientific, Waltham, MA, USA) at 2 d of age, presented in Figure 1. The experimental diets were produced at the North Carolina State University Feed Education Unit (Raleigh, NC, USA) and calculated to be isocaloric. The onion peels were procured commercially from Boardman Foods, Inc. (Boardman, OR, USA). Thereafter, onion peels were manually sorted to remove foreign matter and damaged pieces, washed with potable water, drained, and dried in a single layer at room temperature in an environmentally controlled room until brittle. The dried peels were then vacuum sealed, stored in a dark, dry place, ground into powder, and shipped to Eclessias Extracts (Colorado Springs, CO, USA) for solvent extraction. The experimental diets were provided ad libitum to the chicks throughout the study, formulated to meet or slightly exceed nutritional requirements following guidelines outlined in the Ross broiler nutrition specification handbook [22]. Growth performance indices such as body weight (BW), feed intake (FI), body weight gain (BWG) and feed conversion ratio (FCR) were determined on d 14.

### 2.2. Characterization of Onion Peel Extract

The chemical composition of the OPE powder was determined via independent laboratory analysis by International RINP, Inc. (Laguna Hills, CA, USA). The total polyphenols and quercetin content were quantified using High Performance Liquid Chromatography with Ultraviolet detection (HPLC-UV) to ensure the standardization of the bioactive constituents within the extract lot used throughout the study.

### 2.3. Cecal and Spleen Samples Collection

On 5 and 12 day post infection (dpi), 6 chicks were randomly selected per treatment group and euthanized via CO_2_ asphyxiation. Ceca samples were aseptically removed, placed in sterile bags on ice, and transported to the laboratory for immediate SE enumeration, with results expressed as Log_10_ CFU/g cecal contents. Also, spleen samples were aseptically collected on ice and immediately placed into 4.0 mL cryogenic tube, quickly frozen in liquid nitrogen and stored at −80 °C for subsequent metabolomics analysis.

### 2.4. Sample Preparation

The spleen samples (20 ± 1 mg) were weighed into 1.5 mL Eppendorf tubes then placed on dry ice, and 400 µL of methanol: water (80:20) solution (precooled to −80 °C overnight) was added. Each sample was spiked with two quality control standards, including 10 µL Waters QC Reference Standard (Catalogue number:186006963, Waters, Milford, MA, USA) and 20 µL of a 1:1 mixture of QRESS 1 and QRESS 2 (Catalogue number: MSK-QRESS-KIT, Cambridge Isotope Laboratories, Inc., Tewksbury, MA, USA). A 4 mm stainless steel bead was added to each tube. Samples were briefly vortexed, then homogenized using a 2010 Geno/Grinder (SPEX SamplePrep, Metuchen, NJ, USA) at 1500 rpm in two 30 s cycles. The Geno/Grinder tube holders were precooled in the freezer to keep the samples cold. In the next step, the samples were incubated at −80 °C for 15 min, then placed on wet ice, vortexed for 10 s, and centrifuged at 15,000 rcf for 15 min at 4 °C. A total of 450 µL of the supernatants were transferred into clean 0.5 mL Eppendorf tubes. The supernatants were vortexed, then two 200 µL aliquots were transferred into new 0.5 mL tubes. Pooled QC samples were prepared by pipetting 10 µL from the remainder of the supernatants of all study samples. All samples were evaporated to dryness in a vacuum concentrator (SPD120, Thermo Fisher Scientific, Waltham, MA, USA) maintained at 35 °C. On the day of analysis, one of the two sample aliquots were reconstituted in 50 µL water/acetonitrile (95:5) for reversed-phase liquid chromatography (RPLC), while the second aliquots were reconstituted in 50 µL acetonitrile/water (2:1) for hydrophilic interaction chromatography (HILIC) analysis. Processing blanks were also prepared with every batch of samples.

### 2.5. Chromatographic Conditions

All LC-MS experiments were done on a Thermo Fisher Scientific Vanquish ultra-high performance (UHP) LC (Vanquish Flex™ UHPLC system, Germering, Germany). RPLC analyses were performed on a Waters Acquity Premier BEH C18 (100 × 2.1 mm, 1.7 μm particle size) column. Mobile phase A was 0.1% formic acid in water: acetonitrile (95:5) and mobile phase B was 0.1% formic acid in acetonitrile. The chromatographic gradient was: 0 min (0% B), 1 min (0% B), 18.0 min (100% B), 22.0 min (100% B), 22.1 min (0% B), 25.0 min (0% B). The column oven was set to 40 °C, the flow rate was 0.3 mL/min, and the injection volume was 2 μL. HILIC analyses were performed on a Waters Atlantis Premier BEH Z-HILIC (100 × 2.1 mm, 1.7 μm particle size) zwitterionic column. Mobile phase A was 10 mM ammonium acetate in water adjusted to pH = 10.0 (± 0.1) with ammonium hydroxide, mobile phase B consisted of 10 mM ammonium acetate in 9:1 (*v*/*v*) acetonitrile:water and contained the same amount of ammonium hydroxide as mobile phase A. The chromatographic gradient was: 0 min (100% B), 1 min (100% B), 12.0 min (50% B), 13.0 min (50% B), 13.1 min (100% B), 19.0 min (100% B). The column oven was set to 30 °C, the flow rate was 0.4 mL/min, and the injection volume was 2 μL.

### 2.6. Mass Spectrometric Conditions

Samples were analyzed on a Thermo Fisher Scientific mass spectrometer (Orbitrap ID-X Tribrid, San Jose, CA, USA) in positive and negative ionization modes in separate injections. The heated electrospray ionization source was set to 3500 V and 2500 V in positive and negative mode, respectively. The sheath, aux and sweep gases were 50, 10 and 1 (arb), respectively. The ion transfer tube and vaporizer temperatures were 300 and 350 °C, respectively. The expected LC peak width was set to 6 s. Mild trapping was turned off. Internal mass calibration was achieved with EASY-IC. MS1 resolving power was 120,000. MS2 scans were acquired in the orbitrap with a resolving power of 30,000 using both HCD and CID in a Top 4 analysis with dynamic exclusion for 2.5 s after selecting the same precursor twice within 1 s.

### 2.7. Data Processing and Analysis

Data was analyzed using Compound Discoverer v3.3. The 4 data sets (RPLC+, RPLC−, HILIC+, HILIC−) were all analyzed separately. The analysis workflow included pooled QC correction and the following search nodes for metabolite identification: (a) mzVault with an in-house spectral library containing over 700 common metabolites, as well as the NIST 2023 spectral library, (b) the mzCloud spectral library and c) ChemSpider databases (BioCyc, ChEBI, HMDB, KEGG). The results were exported and the RPLC and HILIC datasets from the same polarity were combined for further analyses.

### 2.8. Statistical Analysis

Growth performance data were analyzed using one-way ANOVA. Microbiological data were evaluated using a Student’s *t*-test to compare the CON-SE and OPE-SE groups, as no colonies were detected in the CON and OPE treatments. Statistical significance was set at *p* < 0.05. The spleen metabolome data were analyzed using MetaboAnalyst v6.0 web-based software (https://www.metaboanalyst.ca/). Differentially abundant metabolites (*p* < 0.05 and absolute fold change (FC) ≥ 2.0 equivalent to |log_2_FC| > 1.0) were determined using a volcano plot analysis. Principal component analysis (PCA) and orthogonal partial least squares discriminant analysis (OPLS-DA) score plots were created to illustrate variation in the spleen metabolome between the experimental treatments. The variable importance in projection (VIP) scores (VIP score > 1.0) were acquired from OPLS-DA results. To elucidate the distinct metabolic pathways in the spleen, a Kyoto Encyclopedia of Genes and Genomes (KEGG) enrichment analysis of the metabolome was conducted. The analysis identified metabolic pathways that differed significantly between chicks fed OPE diets, whether infected or uninfected with SE. The enrichment analyses were performed in the MetaboAnalyst 6.0 platform utilizing *Gallus gallus* library, and the pathways at -log10 (*p*) ≥ 1.3 (equivalent to *p* < 0.05) were considered altered. Furthermore, to visualize the enriched metabolic pathways of spleen metabolites in the experimental groups of the broiler chicks, we employed MetScape v3.1.3 (http://metscape.med.umich.edu/) [23], a plug-in for Cytoscape v3.10.3 (https://cytoscape.org/) [24] to visualize the metabolic compound–enzyme–gene network interaction. The construction of the metabolic network pathway analysis is based on the KEGG and Edinburgh Human Metabolic Network (EHMN) databases. It identified key hub metabolites and their interactions in biological pathways.

## 3. Results

### 3.1. Chemical Composition of Onion Peel Extract

The phytochemical characterization confirmed that the OPE is a highly concentrated polyphenolic fraction. Based on the independent analysis by International RINP, Inc., the total polyphenols and quercetin content were determined to be 64.19% and 4.57%, respectively (Table 2). These values establish the high purity and standardized bioactive profile of the extract used for the experimental trial.

### 3.2. Growth Performance and Cecal SE Concentration in Broiler Chicks

**Figure 2:**
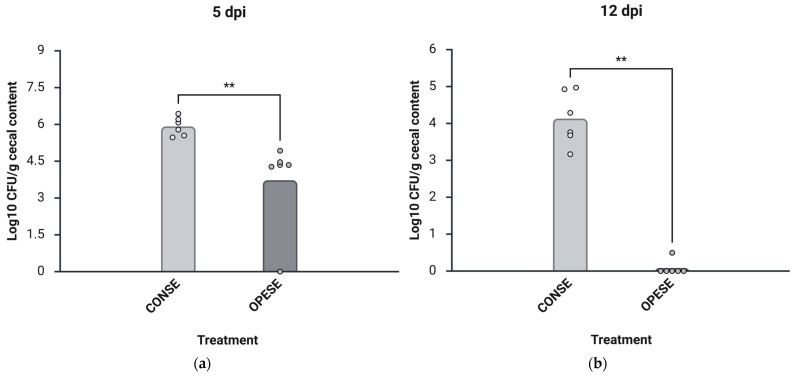
Influence of dietary onion peel extract on the cecal *Salmonella* Enteritidis colonization in broiler chicks. (**a**) 5 dpi (**b**) 12 dpi. ** indicates statistical significance at *p* < 0.05. CON and OPE groups had no detectable SE colonies.

The results showed that birds had similar (*p* > 0.05) growth performances such as BW, FI, BWG and FCR as presented in Table 3. At 5 and 12 dpi, SE colonization was lower in the OPE-SE group than in the CON-SE group (*p* < 0.05). In contrast, no SE was detected in the CON or OPE treatments (Figure 2).

### 3.3. PCA and OPLS-DA Plots

**Figure 3:**
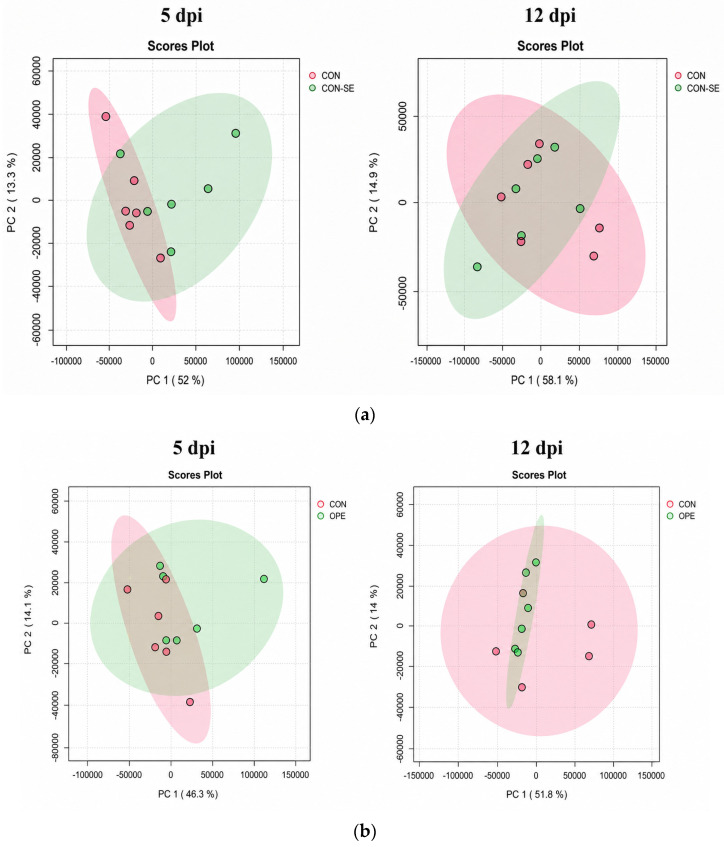

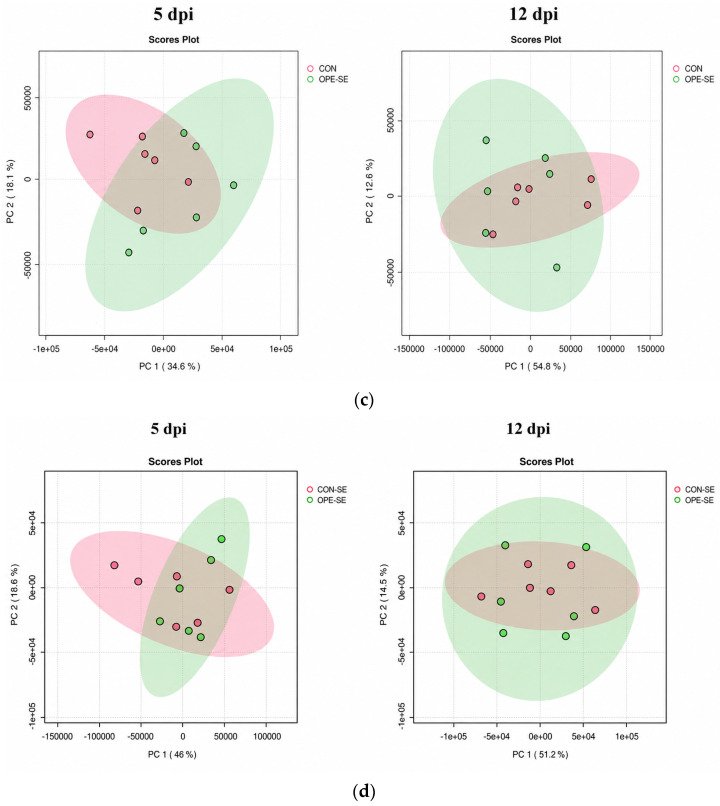
Principal component analysis (PCA) plots (**a**) CON and CON-SE; (**b**) CON and OPE; (**c**) CON and OPE-SE; (**d**) CON-SE and OPE-SE on 5 dpi and 12 dpi of spleen metabolome.

**Figure 4:**
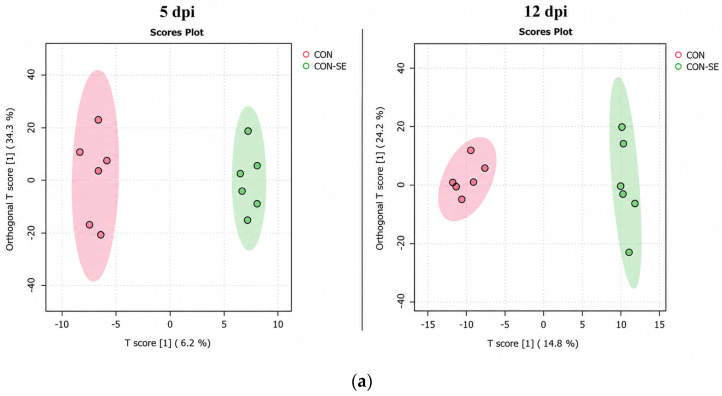

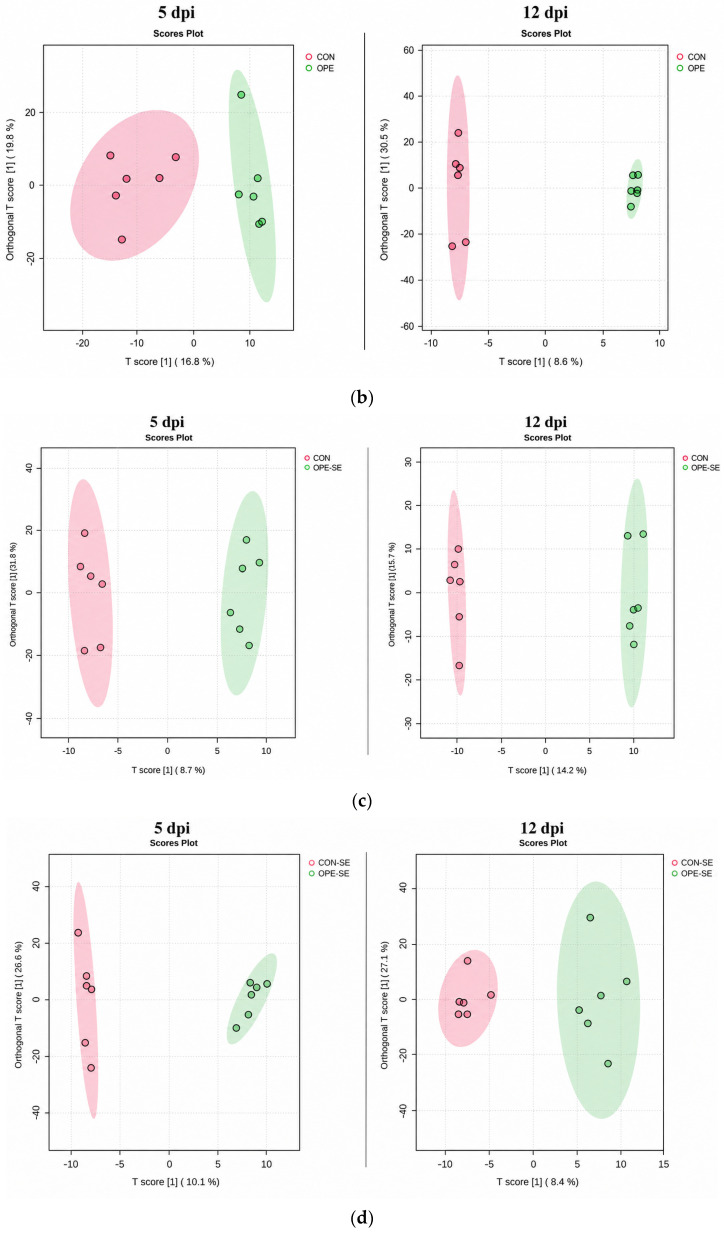
Discriminant analysis by orthogonal partial least square (OPLS-DA) score plots (**a**) CON and CON-SE; (**b**) CON and OPE; (**c**) CON and OPE-SE; (**d**) CON-SE and OPE-SE on 5 dpi and 12 dpi of spleen metabolome.

In this study, untargeted metabolomics was employed to analyze the metabolite profiles in the spleen metabolome in the experimental groups. A total of 857 metabolites were identified in the chicks’ spleen across all the experimental groups. On 5 dpi, PCA plots showed partial overlaps between CON vs. CON-SE, CON vs. OPE, CON vs. OPE-SE, and CON-SE vs. OPE-SE, whereas on 12 dpi, PCA plots showed overlaps between most compared groups except CON vs. CON-SE, which still showed only partial overlap (Figure 3a–d). In contrast, OPLS-DA plots displayed distinct separation between CON and all experimental groups at both 5 dpi and 12 dpi (Figure 4a–d). These results indicate that the overall splenic metabolic profile of CON is distinct from all *Salmonella*-challenged groups (with or without OPE inclusion) at both time points, and that infection alone gradually alters the metabolome over time, as reflected by the changing CON vs. CON-SE relationship from 5 to 12 dpi.

### 3.4. Differential Abundant Metabolites

**Figure 5:**
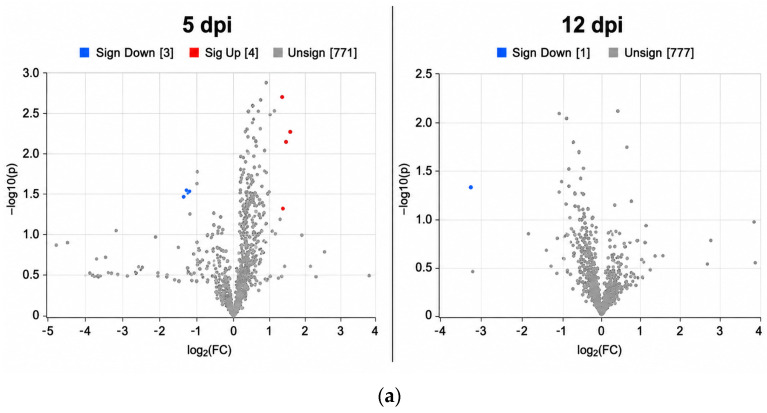

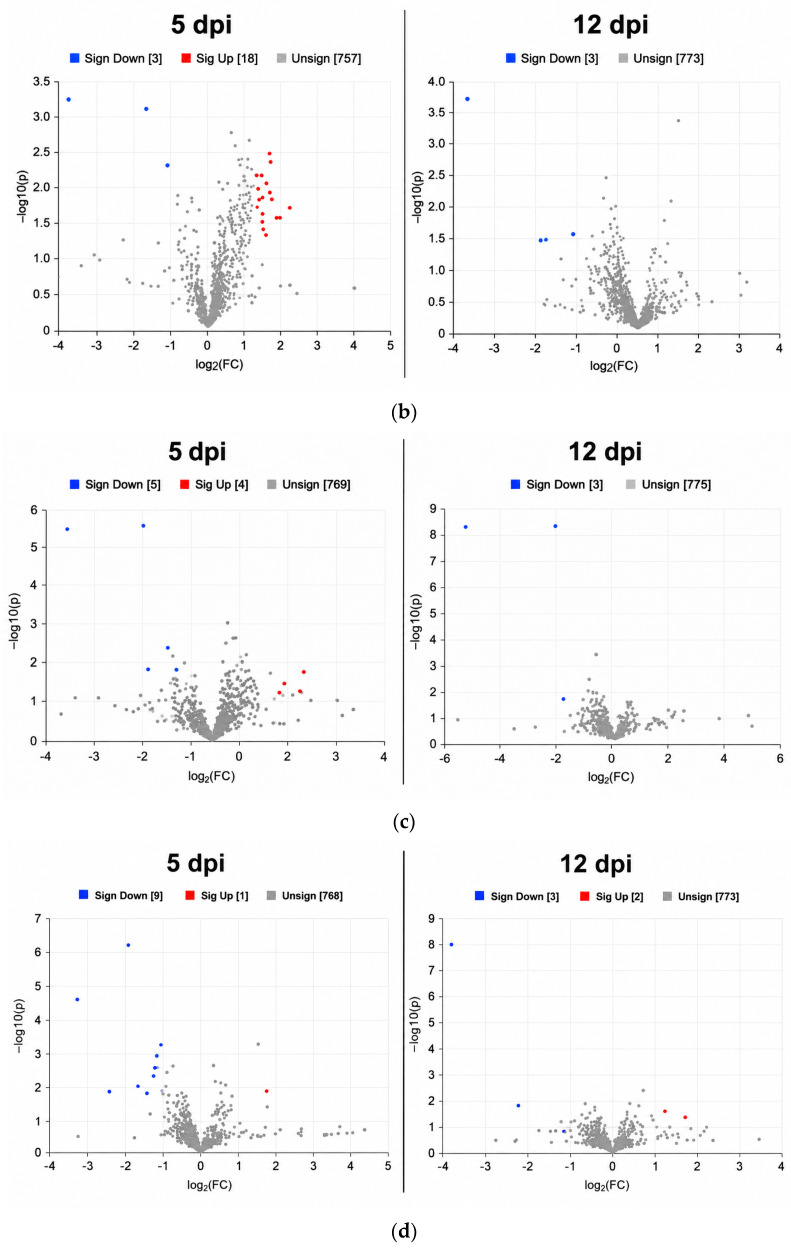
Volcano plot of spleen metabolome (**a**) CON and CON-SE; (**b**) CON and OPE; (**c**) CON and OPE-SE; (**d**) CON-SE and OPE-SE on 5 dpi and 12 dpi of spleen metabolome showing the number of differentially abundant metabolites (*p* < 0.05; FC ≥ 2.0; VIP score > 1.0). Each point in the volcano plot represents a metabolite with blue points representing downregulated differential metabolites, red points representing upregulated differential metabolites, and gray points representing detected metabolites but show no significant differences.

In CON and CON-SE, the results of the volcano plot analysis revealed a total of seven and one differentially abundant (*p* < 0.05; absolute fold change (FC) ≥ 2.0; VIP score > 1.0) metabolites on 5 dpi and 12 dpi, respectively (Figure 5a; see Appendix A, Table A1). Metabolites such as 6-phosphogluconic acid, methylmalonic acid, and propionic acid, were upregulated (*p* < 0.05), while 13,14-dihydro-15-keto-prostagladin D2 was downregulated (*p* < 0.05) on 5 dpi, whereas kynurenic acid was downregulated (*p* < 0.05) on 12 dpi in the CON-SE group (Table 4). The results of the volcano plot analysis revealed that a total of twenty-one and five differentially abundant (*p* < 0.05; FC ≥ 2.0; VIP score > 1.0) metabolites were identified between CON and OPE groups on 5 dpi and 12 dpi, respectively (Figure 5b; see Appendix A, Table A1). On 5 dpi, compared to the CON, the metabolites which include Gly-Tyr, L-Ile-L-Leu, N-Tryptophylglycine, L-Val-L-Met, were upregulated (*p* < 0.05), whereas 3,6-di-O-methyl-beta-D-glucose, 3-O-beta-D-galactosyl-sn-glycerol, were downregulated (*p* < 0.05) on 5 dpi and 12 dpi, and in addition, S-beta-D-glucosyl-L-cysteine was also downregulated (*p* < 0.05) on 12 dpi in OPE group (Table 4). Furthermore, in CON and OPE-SE, the volcano plot analysis revealed that a total of nine and three differentially abundant (*p* < 0.05; FC ≥ 2.0; VIP score > 1.0) metabolites on 5 dpi and 12 dpi were identified, respectively (Figure 5c; see Appendix A, Table A1). Thus, metabolites such as L-Val-Gly, Gly-Gly-Leu, L-Leu-Gly, were upregulated (*p* < 0.05), while 3,6-di-O-methyl-beta-D-glucose and 3-O-beta-D-galactosyl-sn-glycerol, like in OPE group, were downregulated (*p* < 0.05) on 5 dpi and 12 dpi; however, 5-Hydroxy-3-indoleacetic acid and S-beta-D-glucosyl-L-cysteine were also downregulated (*p* < 0.05) on 5 dpi and 12 dpi, respectively in the OPE-SE group (Table 4). Additionally, the volcano plot analysis revealed a total of ten and five differentially abundant (*p* < 0.05; FC ≥ 2.0; VIP score > 1.0) metabolites in CON-SE and OPE-SE on 5 dpi and 12 dpi were identified, respectively (Figure 5d; see Appendix A, Table A1). On 5 dpi and 12 dpi, 3-O-beta-D-galactosyl-sn-glycerol, and 3,6-di-O-methyl-beta-D-glucose were downregulated (*p* < 0.05), while on 5 dpi, propionic acid was downregulated (*p* < 0.05); however, on 12 dpi, pinolenic acid was upregulated (*p* < 0.05), while glycerol-3-phosphate was downregulated (*p* < 0.05).

### 3.5. Enrichment Analysis of Differentiated Metabolites

**Figure 6:**
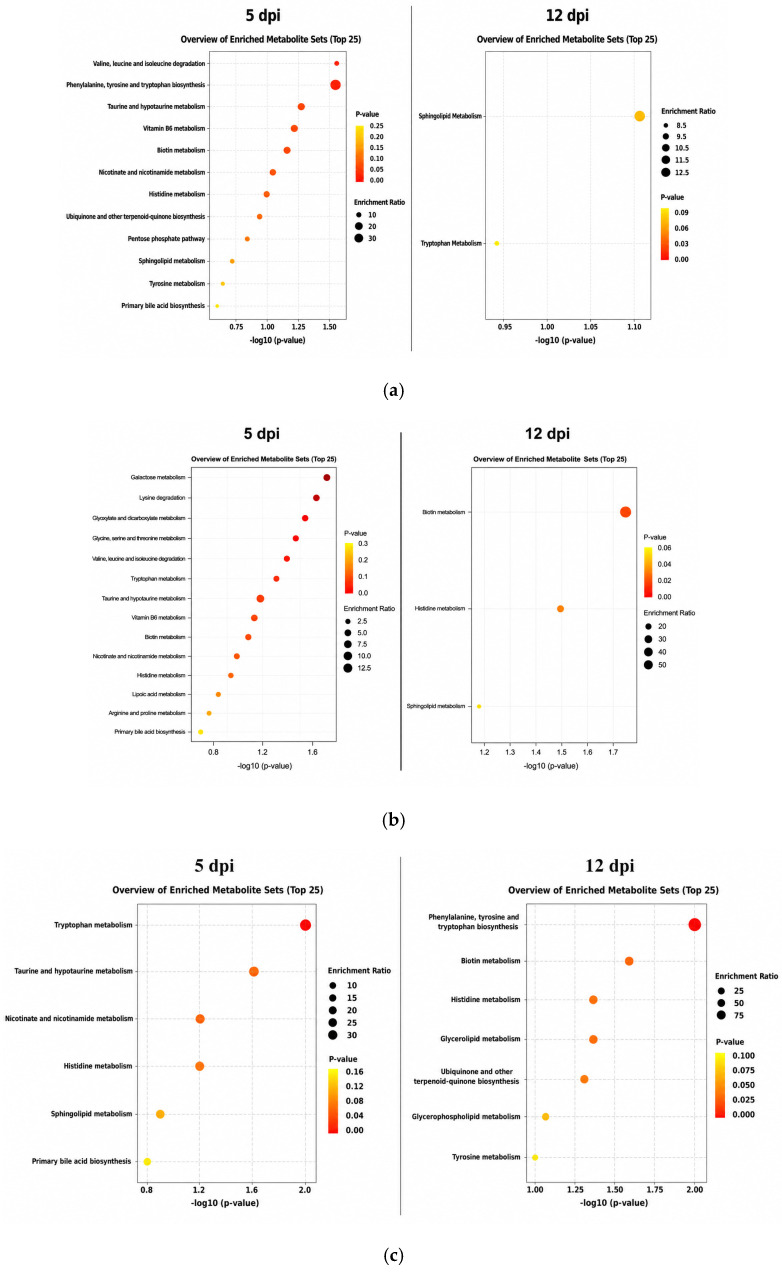

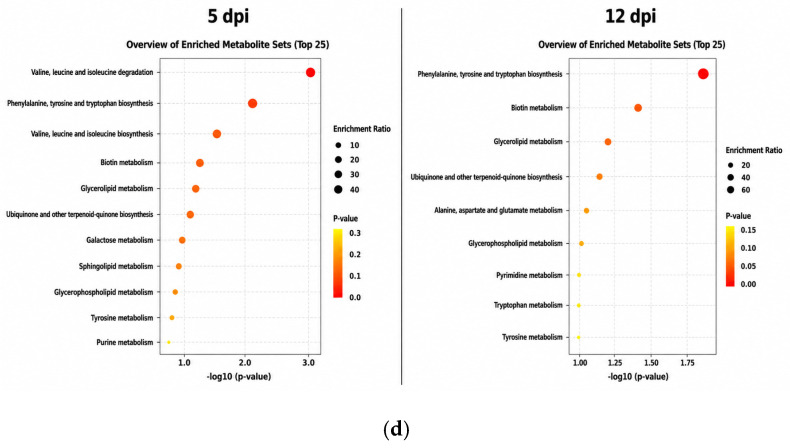
KEGG enrichment analysis (**a**) CON and CON-SE; (**b**) CON and OPE; (**c**) CON and OPE-SE; (**d**) CON-SE and OPE-SE on 5 dpi and 12 dpi of spleen metabolome. Pathways were considered altered when enrichment significance met the threshold of –log_10_(*p*) ≥ 1.3 (equivalent to *p* < 0.05).

KEGG enrichment analysis results revealed that spleen metabolites of the broiler chicks in the CON-SE group enriched (*p* < 0.05) the following pathways: valine, leucine and isoleucine degradation; and phenylalanine, tyrosine and tryptophan biosynthesis on 5 dpi, whereas no significant enrichment (*p* > 0.05) was observed on 12 dpi (Figure 6a; Table 5). On 5 dpi, the results of the enrichment analysis of the differentially abundant metabolites showed that OPE group enriched (*p* < 0.05) galactose metabolism; lysine degradation; glyoxylate and dicarboxylate metabolism; glycine, serine and threonine metabolism; and valine, leucine and isoleucine degradation; while OPE-SE group enriched (*p* < 0.05) the following pathways: tryptophan metabolism; and taurine and hypotaurine metabolism. By 12 dpi, both OPE and OPE-SE groups enriched (*p* < 0.05) biotin metabolism; and histidine metabolism pathways. Additionally, OPE-SE group enriched (*p* < 0.05) phenylalanine, tyrosine and tryptophan biosynthesis; glycerolipid metabolism; and ubiquinone and other terpenoid-quinone biosynthesis on 12 dpi. Between CON-SE and OPE-SE, pathways such as valine, leucine and isoleucine degradation; and phenylalanine, tyrosine and tryptophan biosynthesis were enriched (*p* < 0.05) on 5 dpi, while phenylalanine, tyrosine and tryptophan biosynthesis and biotin metabolism pathways were enriched (*p* < 0.05) on 12 dpi (Figure 6b–d; Table 5).

### 3.6. Metabolic Network Interaction

**Figure 7:**
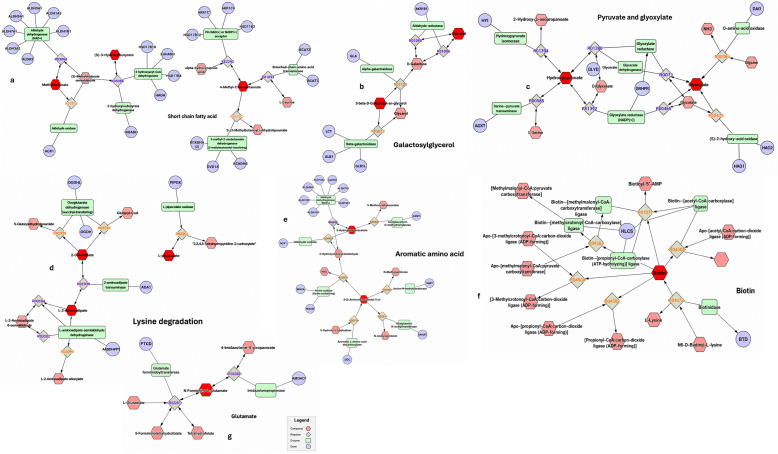
Metabolic network interaction analysis was performed using the MetScape 3 App for Cytoscape. Node colors and shapes indicate different molecules. (**a**) Short-chain fatty acid network; (**b**) Galactosylglycerol network; (**c**) Pyruvate and glyoxylate network; (**d**) Lysine degradation network; (**e**) Aromatic amino acid network; (**f**) Biotin network; (**g**) Glutamate network.

Furthermore, to visualize the metabolic pathways from the spleen metabolites, MetScape was utilized to construct metabolic networks including the interplay between metabolites and genes, compound networks and provide information for reactions, enzymes and associated pathways. It analyzed metabolic networks using an internal database that integrates data from KEGG and EHMN. Several metabolic networks were observed across the treatment groups. In the CON-SE group, network interaction analysis revealed that the short-chain fatty acid metabolic network was affected by the SE infection in the broiler chicks devoid of dietary OPE intervention. Additionally, dietary OPE fed to the broiler chicks that were not infected with SE, was observed to greatly influence metabolic networks such as pyruvate and glyoxylate, pipecolate and oxoadipate, galactosylglycerol, lysine degradation, and glutamate in the OPE group. Meanwhile, OPE intervention in the broiler chicks infected with SE was observed to greatly impact aromatic amino acids and biotin metabolic networks in the OPE-SE group, as shown in Figure 7.

## 4. Discussion

In this study, an untargeted metabolomics approach was used to elucidate the immune mediating capacity of the spleen in *Salmonella*-infected broiler chicks fed an OPE-supplemented diet. The observed growth performance of the present study in SE-challenged and OPE-supplemented groups was not significant. This aligns with previous reports that phytobiotic additives can produce variable growth responses in *Salmonella*-challenged broilers, likely due to differences in challenge severity, duration, and bioactive composition [25]. Dietary OPE reduced cecal SE colonization throughout this study, suggesting that the concentrated quercetin (4.57%) and high density of other polyphenols (64.19%) identified in the OPE powder contributed to disrupting bacterial cecal colonization [26]. These phytochemical levels acted as natural antimicrobial agents, improving gut health by modulating the microbiota, and strengthening intestinal barrier integrity [27,28] by limiting pathogen proliferation in the gut of the chicken. The level of these bioactive compounds may have limited *Salmonella* adhesion, invasion, and subsequent systemic proliferations. Overall, OPE supplementation provided the necessary biochemical environment to support a robust immune regulatory response in the spleen metabolome against *Salmonella* infection compared to the CON diet.

The distinct OPLS-DA separation and evolving PCA overlaps between the control and treatment groups indicate that both *Salmonella* and OPE induce discrete, time-dependent metabolic shifts in the spleen. While partial overlaps at 5 dpi suggest a shared acute metabolic stress response, the distinct clustering at 12 dpi reflects a systemic reprogramming of splenic pathways likely involving immune metabolism by OPE intervention and infection progression.

Several key metabolites were elevated in the spleen of SE-infected chicks that did not receive OPE intervention, including 6-phosphogluconic acid, methylmalonic acid, and propionic acid. Previous studies indicate that 6-phosphogluconic acid and methylmalonic acid are intermediates of the pentose phosphate pathway and propionate metabolism, respectively, contributing to glucose utilization and entry into the tricarboxylic acid (TCA) cycle during metabolic stress [29,30]. Propionic acid also plays a major role in short-chain fatty acid metabolism and is produced by gut microbial activity, where it disrupts pathogenic bacterial membrane integrity and intracellular pH balance [31]. The increase in these metabolites reflects the establishment of SE infection and a natural metabolic adaptation aimed at limiting bacterial proliferation; however, without a targeted intervention, this response may be insufficient or unsustainable. In contrast, 13,14-dihydro-15-keto-prostagladin D2 and kynurenic acid decreased at different stages of SE infection. Prostaglandins promote acute inflammation by recruiting phagocytes and enhancing local immune responses against extracellular pathogens [32]. Kynurenic acid, a metabolite of the tryptophan–kynurenine pathway, plays a critical role in limiting excessive inflammation and oxidative damage during infection [33,34]. The reduction in these metabolites suggests a diminished or dysregulated immune response, impairing the chicks’ ability to control inflammation and oxidative stress. This pattern corresponds with the higher cecal SE colonization observed in the CON group compared with chicks receiving the OPE diet, further indicating that OPE supplementation supports a more effective immune metabolic response during *Salmonella* challenge.

Dietary OPE increased the abundance of Gly-Tyr, L-Ile-L-Leu, N-Tryptophylglycine, and L-Val-L-Met in the spleen of broiler chicks without SE infection. Gly-Tyr is a bioactive dipeptide known to reduce inflammation and suppress pro-inflammatory cytokine production [35]. It also serves as a precursor for catecholamines that regulate immune cell activity and enhance reactive oxygen species (ROS) generation, supporting effective pathogen clearance [35,36]. L-Ile-L-Leu has been shown to enhance humoral immunity by increasing IgA and IgG levels, pathogen-specific antibody production, and cytokine regulation in piglets [37]. Its *in ovo* administration also improved spleen recovery following chronic heat stress in broilers, indicating enhanced immune competence [38]. N-Tryptophylglycine is an emerging peptide associated with immune modulation, improved disease resistance, and regulation of immune cell activity [39,40], and it contributes to glutathione (GSH) synthesis [36]. L-Val-L-Met supports gut and immune function through its valine component, which improves duodenal villus height, jejunal development, and spleen lymphocyte proliferation in chickens [41,42]. Methionine further serves as a key precursor for GSH, the substrate required for glutathione peroxidase, both essential for maintaining redox balance and protecting tissues from oxidative damage [43,44]. The generation of these metabolites suggests that dietary OPE can enhance immune cell protection against oxidative damage while supporting antimicrobial defense. This aligns with evidence that plant-based polyphenols neutralize ROS, prevent oxidative damage, and disrupt bacterial membranes, leading to cytoplasmic leakage and reduced pathogen viability [45,46,47]. Thus, OPE may help mitigate oxidative stress and inflammation, contributing to improved resilience under potential pathogen exposure or physiological stress. S-beta-D-glucosyl-L-cysteine, a sulfur containing *Allium* metabolite, was reduced in OPE-fed chicks without SE infection, indicating altered sulfur amino acid metabolism. Similar shifts have been reported in broilers receiving onion extract or sulfur containing supplements under stress, where changes in cysteine-related metabolism were associated with modified oxidative status and growth responses [48,49].

Furthermore, dietary OPE increased L-Val-Gly, Gly-Gly-Leu, and L-Leu-Gly in the spleen of SE-infected chicks, indicating that OPE modulates peptide and amino acid metabolism during *Salmonella* infection. This agrees with evidence that polyphenol-bound metabolites restore amino acid pathways, including valine-leucine-isoleucine biosynthesis [50]. The elevated small peptides reflect enhanced proteolysis and protein turnover associated with immune activation. OPE may also improve intestinal absorption or reduce catabolism of branched-chain amino acids, increasing their availability for immune cell proliferation and protein synthesis. Gly-Gly-Leu and related dipeptides promote cell proliferation and gut barrier integrity [51], supporting colonization resistance and limiting pathogen translocation [52]. Glycine residues from these peptides may further contribute to glutathione synthesis, enhancing antioxidant capacity during *Salmonella*-induced oxidative stress [53]. Because *Salmonella* infection increases glycolysis and suppresses the TCA cycle [54], OPE may help maintain metabolic balance by preventing excessive depletion of amino acid pools. Collectively, these patterns suggest that OPE polyphenols rich in quercetin support nutrient utilization, metabolic homeostasis, and immune function during infection. Pinolenic acid was upregulated in OPE-fed, SE-challenged chicks, indicating modulation of omega-6 fatty acid metabolism. Its known anti-inflammatory and immunomodulatory properties [55] suggest that OPE may promote a controlled inflammatory response that enhances pathogen clearance while limiting tissue damage, consistent with its protective effects during SE infection. Conversely, 3,6-di-O-methyl-beta-D-glucose and 3-O-beta-D-galactosyl-sn-glycerol decreased in OPE-fed chicks, regardless of infection status. As intermediates of galactose and glycerolipid metabolism, respectively [56,57], these metabolites contribute to glycolytic support, lipid signaling, and energy storage. Their reduction may reflect OPE-mediated restraint of excessive glycolysis or lipid derived signaling, promoting a balanced immune response. Previous reports suggest that polyphenols also interact with gut microbes to generate bioactive metabolites, including short-chain fatty acids that support immune and epithelial energy needs [58], reducing the energy burden associated with inflammation [59,60]. The decrease in 5-hydroxy-3-indoleacetic acid, the primary serotonin metabolite, is notable given its role in regulating inflammation through the kynurenine-aryl hydrocarbon receptor pathway [61]. Polyphenols influence tryptophan metabolism [48,62], and its reduction suggests that OPE may help restore immune homeostasis by reducing reliance on serotonin-mediated anti-inflammatory compensation. Overall, these metabolic shifts indicate that dietary OPE supports a regulated inflammatory response and metabolic stability during *Salmonella* infection, consistent with the reduced cecal SE colonization observed in OPE-fed chicks.

Enrichment analysis in this study revealed that valine, leucine, and isoleucine degradation, along with phenylalanine, tyrosine, and tryptophan biosynthesis, were altered in *Salmonella*-infected broiler chicks without dietary OPE interventions. Branched-chain amino acids (BCAAs) and their degradation pathways are essential for shaping immune responses, and their increased catabolism reflects a metabolic shift toward meeting elevated energy and immune demands during infection, prioritizing defense over growth-related anabolic processes [63], which may reflect similar growth performances obtained in this study. Likewise, aromatic amino acid pathways, phenylalanine, tyrosine, and tryptophan modulate inflammation, immune cell activity, and host–microbe interactions [64], indicating an adaptive response to support the synthesis of neurotransmitters and immunomodulators required for effective innate immunity during SE infection. In non-infected chicks, dietary OPE enhanced galactose metabolism; lysine degradation; glyoxylate and dicarboxylate metabolism; glycine, serine, and threonine metabolism; and BCAA degradation. Galactose metabolism supplies glucose-1-phosphate for early energy needs, glycogen storage, and glycoprotein synthesis [65]. Lysine degradation generates acetyl-CoA for the TCA cycle [66,67], while the glyoxylate and dicarboxylate pathway supports amino acid catabolism, redox balance, and intermediary metabolism under stress [68]. Glycine, serine, and threonine metabolism contribute to nucleotide synthesis, antioxidant defense, and immune function [69]. BCAA degradation yields intermediates such as acetyl-CoA, succinyl-CoA, and acetoacetate, which fuel energy production and modulate immunity [70]. Altogether, these pathways indicate that OPE can enhance energy efficiency, antioxidant capacity, and immune readiness even in the absence of infection. Furthermore, in *Salmonella*-infected chicks, dietary OPE altered tryptophan metabolism and taurine and hypotaurine metabolism. Tryptophan metabolism regulates immune balance through serotonin and kynurenine production [71], while taurine and hypotaurine metabolism supports antioxidant defense, membrane stability, and calcium-dependent immune signaling [72]. Dietary OPE enhanced phenylalanine, tyrosine, and tryptophan biosynthesis; glycerolipid metabolism; and ubiquinone and terpenoid-quinone biosynthesis. These pathways support neurotransmitter production, lipid-based immune signaling, mitochondrial energy generation, and antioxidant protection during immune challenge [73,74,75,76]. Additionally, OPE promoted biotin and histidine metabolism in both infected and non-infected chicks. Biotin supports energy production, amino acid metabolism, and cellular integrity [77], while histidine metabolism contributes to protein synthesis, histamine production, and antioxidant defense [78]. Therefore, these findings suggest that dietary OPE may enhance immune regulatory and energy producing pathways, promoting resilience, and metabolic homeostasis in both *Salmonella*-infected and non-infected broiler chicks.

**Figure 8:**
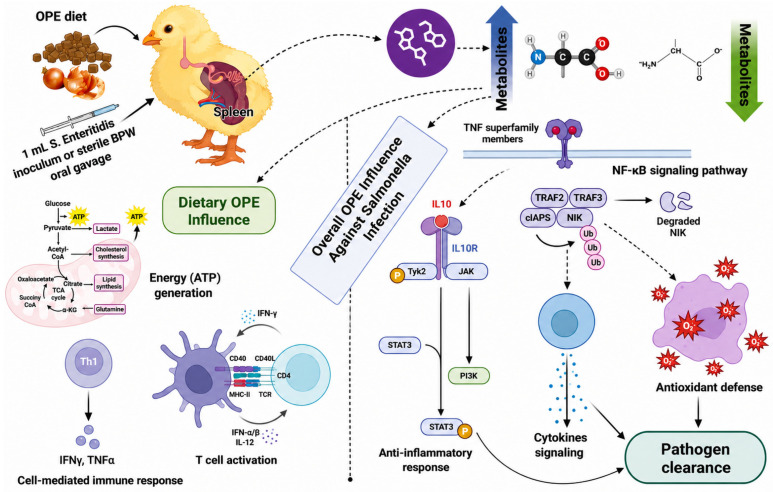
Diagram illustrating the onion-peel-extract-mediated modulation of the spleen metabolome in *Salmonella*-infected broiler chicks. Blue arrow pointing up indicates an increase, whereas the green arrow pointing down denotes a decrease in abundant metabolites. Created in BioRender. Iwuozo, O. (2026) https://BioRender.com/rkh8o3a.

The metabolic network interaction analysis revealed that SE infection perturbed the short-chain fatty acid (SCFA) metabolic network in chicks not receiving dietary OPE. SCFAs are essential regulators of gut health, immune function, and energy metabolism, acting as key signaling molecules for enterocytes [79,80]. Thus, the disruption of SCFA-related networks reflects a natural metabolic adaptation to SE infection. In contrast, dietary OPE in non-infected chicks influenced pyruvate and glyoxylate, pipecolate and oxoadipate, galactosylglycerol, and glutamate-associated networks. These metabolic networks support mitochondrial energy production, oxidative stress regulation, cellular and immune function, and intestinal health [81,82,83], while also affecting nitrogen balance through the lysine degradation metabolic network [84]. This pattern suggests that OPE may enhance cellular energy balance and antioxidant capacity which can promote mitochondrial function and amino acid turnover, thereby supporting gut integrity and immune preparedness in broiler production systems [85]. Furthermore, in *Salmonella*-infected chicks, dietary OPE modulated aromatic amino acid and biotin metabolic network interactions. These metabolic networks are closely linked to immune regulation, energy homeostasis, antioxidant defense, and gut–brain axis communication [73,86,87]. Accordingly, the dietary OPE intervention in this study benefited the *Salmonella*-infected broiler chicks by modulating metabolic mechanisms capable of maintaining immune response, antioxidant capacity, and improving pathogen clearance, and enhancing energy efficiency and immune readiness in chicks without *Salmonella* infection (Figure 8).

## 5. Conclusions

Untargeted spleen metabolomics revealed key metabolites and pathways associated with the broiler chicks’ response to *Salmonella* infection without dietary OPE intervention. In non-infected chicks, OPE influenced pathways linked to amino acid turnover and energy metabolism, suggesting a role in maintaining metabolic readiness prior to pathogen exposure. During SE challenge, OPE modulated amino acid, peptide, lipid, and energy-related metabolic networks, including tryptophan-associated metabolites and aromatic amino acid–biotin interactions, reflecting changes in immune metabolic adaptations that aligned with reduced cecal SE colonization. Therefore, these metabolomic signatures demonstrate that dietary OPE modulated spleen immune metabolism in ways consistent with reduced SE colonization. These findings highlight OPE as a promising natural antimicrobial, that is capable of modulating the metabolic pathways in the chicken in a manner that heightens immune response against *Salmonella* infections, thereby supporting its potential application in poultry production and food safety.

Future research may consider conducting experiments that will involve performing functional assays of significant metabolites that belong to the pathways altered in this study, particularly those associated with tryptophan metabolism, aromatic amino acid interactions, and biotin-linked networks. Integrating the result of such functional assays with *Salmonella* colonization dynamics will further clarify the underlying mechanisms of how OPE contributes to host defense mechanisms during infection.

## Figures and Tables

**Table 1:** Experimental diets composition ^1^ (% “as is”).

	Starter Diets ^1^ (d 1–14)	
Ingredients	CON Diet	OPE Diet
Corn (7.5% Crude Protein)	51.46	51.46
Soybean meal (47.5% Crude Protein)	40.39	40.39
Onion peel extract * (g/kg)	-	6.00
Poultry fat	3.64	3.64
Limestone	1.08	1.08
Mono-Dicalcium phosphate	2.03	2.03
Salt NaCl	0.40	0.40
Sodium bicarbonate	0.02	0.02
L-Lysine HCl 98%	0.13	0.13
DL-Methionine 99.0%	0.34	0.34
L-Threonine 98.5%	0.11	0.11
NCSU Poultry Vitamin Premix ^2^	0.05	0.05
NCSU Poultry Mineral Premix ^3^	0.20	0.20
Choline chloride 60%	0.10	0.10
Selenium Premix ^+^	0.05	0.05
Corn (7.5% Crude Protein)	51.46	51.46
Soybean meal (47.5% Crude Protein)	40.39	40.39
Calculated nutrient composition		
Metabolizable energy (Kcal/kg)	3117	3117
Total sulfur amino acids, %	1.03	1.03
Lysine, %	1.42	1.42
Calcium, %	0.96	0.96
Available phosphorus, %	0.48	0.48

^1^ Diets used in the study included the following: (i) unmedicated corn-soybean meal (SBM) basal without OPE (CON diet); (ii) OPE diet in which onion peel extract was incorporated into unmedicated corn-SBM basal at 0.6% (i.e., 6.0 g/kg diet). Each of these two diets were separately formulated for the starter (d 1 to 14) of study. * Onion peel extract was added at 0.6% in OPE diet. ^2^ Mineral Premix, supplied per kilogram of diet: Manganese (Mn), 60 mg; Zinc (Zn), 60 mg; Iron (Fe), 40 mg; Copper (Cu), 5 mg; Iodine (I), 1.2 mg; Cobalt (Co), 0.5 mg. ^3^ Vitamin Premix, supplied per kilogram of diet: Vitamin A (6600 IU), Vitamin D (1980 IU), Vitamin E (33 IU), Vitamin B12 (0.02 mg), Biotin (0.13 mg), Menadione (1.98 mg), Thiamine (1.98 mg), Riboflavin (6.60 mg), d-Pantothenic Acid (11.0 mg), Vitamin B6 (3.96 mg), Niacin (55.0 mg), Folic Acid (1.1 mg). ^+^ Selenium Premix provides 0.3 mg Selenium/Kg of feed as sodium selenite.

**Table 2:** Bioactive composition of the standardized onion peel extract.

Component	Composition (%)	Analysis Method
Total Polyphenols	64.19	HPLC-UV
Quercetin	4.57	HPLC-UV

Extract analyzed by International RINP, Inc., 23151 Verdugo Dr., Suite 101, Laguna Hills, CA 92653, USA.

**Table 3:** Effect of dietary onion peel extract on growth performance of broiler chicks (d 1–14).

Treatment	Body Weight (BW, kg/Bird)	Body Weight Gain (BWG, kg/Bird)	Feed Intake (FI, kg/Bird)	FCR (kg:kg)
CON	0.508	0.420	0.450	1.075
CON-SE	0.478	0.393	0.375	0.980
OPE	0.478	0.393	0.405	1.035
OPE-SE	0.420	0.400	0.380	1.170
Pooled SEM	0.044	0.044	0.036	0.065
*p*-value	0.835	0.965	0.521	0.425

FCR = feed conversion ratio calculated as feed-to-gain ratio and adjusted for mortality by including the gains of dead birds in the calculations.

**Table 4:** Top upregulated or downregulated differential metabolites in spleen metabolome of the experimental groups on 5 dpi and 12 dpi.

Comparison	Time Points	Regulation	S/N	Metabolites	VIP	*p*-Value	Log_2_FC
CON vs. CON-SE	5 dpi	Up	1	6-Phosphogluconic acid	2.103	0.002	1.111
			2	Methylmalonic acid	1.982	0.006	1.344
			3	Propionic acid	1.937	0.008	1.230
		Down	1	13,14-dihydro-15-keto-prostagladin D2	1.694	0.034	−1.352
	12 dpi	Down	1	Kynurenic acid	2.438	0.049	−3.214
CON vs. OPE	5 dpi	Up	1	Gly-Tyr	2.027	0.004	1.219
			2	L-Ile-L-Leu	2.135	0.004	1.227
			3	N-Tryptophylglycine	1.972	0.015	1.100
			4	L-Val-L-Met	1.869	0.015	1.240
		Down	1	3,6-di-O-methyl-beta-D-glucose	2.075	0.001	−3.958
			2	3-O-beta-D-galactosyl-sn-glycerol	2.092	0.001	−2.278
	12 dpi	Down	1	3-O-beta-D-galactosyl-sn-glycerol	3.023	0.000	−1.933
			2	3,6-di-O-methyl-beta-D-glucose	2.988	0.000	−3.884
			3	S-beta-D-glucosyl-L-cysteine	2.168	0.030	−1.143
CON vs. OPE-SE	5 dpi	Up	1	L-Val-Gly	1.779	0.017	1.614
			2	Gly-Gly-Leu	1.550	0.037	1.181
			3	L-Leu-Gly	1.516	0.048	1.194
		Down	1	3-O-beta-D-galactosyl-sn-glycerol	2.535	1.85 × 10^−6^	−2.090
			2	3,6-di-O-methyl-beta-D-glucose	2.473	7.99 × 10^−6^	−3.767
			3	5-Hydroxy-3-indoleacetic acid	1.877	0.014	−1.029
	12 dpi	Down	1	3-O-beta-D-galactosyl-sn-glycerol	3.330	3.50 × 10^−9^	−2.284
			2	3,6-di-O-methyl-beta-D-glucose	3.325	5.05 × 10^−9^	−4.434
			3	S-beta-D-glucosyl-L-cysteine	2.305	0.023	−1.356
CON-SE vs. OPE-SE	5 dpi	Down	1	3-O-beta-D-galactosyl-sn-glycerol 2	2.996	0.000001	−1.727
			2	3,6-di-O-methyl-beta-D-glucose	2.913	0.00002	−2.929
			3	Propionic acid	2.478	0.004	−1.017
	12 dpi	Up	1	Pinolenic acid	2.393	0.019	1.423
		Down	1	3,6-di-O-methyl-beta-D-glucose	3.342	0.00000001	−3.946
			2	3-O-beta-D-galactosyl-sn-glycerol	3.326	0.00000001	−2.062
			3	Glycerol 3-phosphate	2.490	0.015	−2.254

*p*-value < 0.05; VIP, variable importance projection threshold: VIP score > 1.0; dpi, day post infection. Significant metabolites were filtered based on an absolute fold change (FC) ≥ 2.0 equivalent to |log_2_FC| > 1.0, representing a minimum two-fold difference in abundance. Positive values denote upregulation and negative values denote downregulation relative to the control group.

**Table 5:** Top KEGG pathways of differential metabolites in spleen metabolome in the experimental groups on 5 dpi and 12 dpi.

	CON vs. CON-SE		CON vs. OPE		CON vs. OPE-SE		CON-SE vs. OPE-SE	
Time Points	KEGG Pathway	Number of Differential Metabolites	KEGG Pathway	Number of Differential Metabolites	KEGG Pathway	Number of Differential Metabolites	KEGG Pathway	Number of Differential Metabolites
5 dpi	Valine, leucine and isoleucine degradation	39	Galactose metabolism	27	Tryptophan metabolism	41	Valine, leucine and isoleucine degradation	39
	Phenylalanine, tyrosine and tryptophan biosynthesis	4	Lysine degradation	30	Taurine and hypotaurine metabolism	8	Phenylalanine, tyrosine and tryptophan biosynthesis	4
			Glyoxylate and dicarboxylate metabolism	31				
			Glycine, serine and threonine metabolism	33				
			Valine, leucine and isoleucine degradation	39				
12 dpi			Biotin metabolism	10	Phenylalanine, tyrosine and tryptophan biosynthesis	4	Phenylalanine, tyrosine and tryptophan biosynthesis	4
			Histidine metabolism	16	Biotin metabolism	10	Biotin metabolism	10
					Histidine metabolism	16		
					Glycerolipid metabolism	16		
					Ubiquinone and other terpenoid-quinone biosynthesis	18		

Note: dpi, day post infection.

## Data Availability

The original contributions presented in the study are included in the article. Further inquiries can be directed to the corresponding author.

## Abbreviations

The following abbreviations are used in this manuscript:

OPE	Onion peel extract
SE	*Salmonella* Enteritidis
HPLC-MS	High performance liquid chromatography mass spectrometry
HPLC-UV	High performance liquid chromatography with ultraviolet detection
dpi	Day post infection
NA	Nalidixic acid
CFU	Colony forming unit
SBM	Soybean meal
BW	Body weight
FI	Feed intake
BWG	Body weight gain
FCR	Feed conversion ratio
UHPLC	Ultra-high performance liquid chromatography
RPLC	Reversed-phase liquid chromatography
HILIC	Hydrophilic interaction chromatography
PCA	Principal component analysis
OPLS-DA	Orthogonal partial least squares discriminant analysis
VIP	Variable importance in projection
KEGG	Kyoto Encyclopedia of genes and genomes
BioCyc	Biological cycles
ChEBI	Chemical entities of biological interest
HMDB	Human metabolome database
EHMN	Edinburgh human metabolic network
TCA	Tricarboxylic acid
BCAA	Branched-chain amino acid
GSH	Glutathione
ROS	Reactive oxygen species
SCFA	Short-chain fatty acid

## Appendix A

**Table A1:** All differential abundant metabolites in spleen metabolome of the experimental groups on 5 dpi and 12 dpi.

Comparison	Time Points	Regulation	S/N	Metabolites	VIP	*p*-Value	Log_2_FC
CON vs. CON-SE	5 dpi	Up	1	6-Phosphogluconic acid	2.103	0.002	1.111
			2	Methylmalonic acid	1.982	0.006	1.344
			3	Propionic acid	1.937	0.008	1.230
			4	N-Acetylglucosaminitol	1.565	0.048	1.253
		Down	1	13,14-dihydro-15-keto-prostagladin D2	1.694	0.034	−1.352
			2	Bethanidine	1.670	0.031	−1.246
			3	2-(1H-Benzimidazol-2-yl)-3-(5-nitro-2-furyl)acrylonitrile	1.620	0.033	−1.217
	12 dpi	Down	1	Kynurenic acid	2.438	0.049	−3.214
CON vs. OPE	5 dpi	Up	1	Gly-Tyr	2.027	0.004	1.219
			2	L-Ile-L-Leu	2.135	0.004	1.227
			3	N-Tryptophylglycine	1.972	0.015	1.100
			4	L-Val-L-Met	1.869	0.015	1.240
			5	L-Threonyl-L-leucine	1.990	0.007	1.006
			6	(2S)-4-(Methylsulfanyl)-2-{[(2S)-2-pyrrolidiniumylcarbonyl]amino}butanoate	1.987	0.007	1.078
			7	4-Amino-1-(6-aminopurin-9-yl)pyrimidin-2-one	2.079	0.009	1.148
			8	Valylproline	1.909	0.010	1.017
			9	2-(Glycylamino)acetic acid benzyl ester	2.018	0.012	1.206
			10	L-Ser-L-Ala	1.843	0.015	1.053
			11	L-Val-L-Ala	1.760	0.018	1.029
			12	L-Val-Gly	1.836	0.019	1.529
			13	H-GLY-MET-OH	1.848	0.024	1.096
			14	L-Leu-Gly	1.879	0.027	1.371
			15	Gly-Gly-Leu	1.747	0.027	1.318
			16	Asn-Leu	1.824	0.032	1.087
			17	L-Val-L-Thr	1.444	0.040	1.098
			18	2-Methylbutyryl-L-carnitine	1.740	0.048	1.132
		Down	1	3,6-di-O-methyl-beta-D-glucose	2.075	0.001	−3.958
			2	3-O-beta-D-galactosyl-sn-glycerol	2.092	0.001	−2.278
			3	2-(1H-Benzimidazol-2-yl)-3-(5-nitro-2-furyl)acrylonitrile	1.884	0.005	−1.208
	12 dpi	Down	1	3-O-beta-D-galactosyl-sn-glycerol	3.023	0.000	−1.933
			2	3,6-di-O-methyl-beta-D-glucose	2.988	0.000	−3.884
			3	S-beta-D-glucosyl-L-cysteine	2.168	0.030	−1.143
			4	N-(4-Aminobenzoyl)-L-glutamic acid	2.058	0.035	−1.943
			5	Dihydroceramide C2	2.079	0.037	−1.597
CON vs. OPE-SE	5 dpi	Up	1	L-Val-Gly	1.779	0.017	1.614
			2	Gly-Gly-Leu	1.550	0.037	1.181
			3	L-Leu-Gly	1.516	0.048	1.194
			4	Asn-Leu	1.726	0.046	1.027
		Down	1	3-O-beta-D-galactosyl-sn-glycerol	2.535	1.85 × 10^−6^	−2.090
			2	3,6-di-O-methyl-beta-D-glucose	2.473	7.99 × 10^−6^	−3.767
			3	5-Hydroxy-3-indoleacetic acid	1.877	0.014	−1.029
			4	2-(1H-Benzimidazol-2-yl)-3-(5-nitro-2-furyl)acrylonitrile	1.976	0.006	−1.316
			5	Bethanidine	1.852	0.016	−1.341
	12 dpi	Down	1	3-O-beta-D-galactosyl-sn-glycerol	3.330	3.50 × 10^−9^	−2.284
			2	3,6-di-O-methyl-beta-D-glucose	3.325	5.05 × 10^−9^	−4.434
			3	S-beta-D-glucosyl-L-cysteine	2.305	0.023	−1.356
CON-SE vs. OPE-SE	5 dpi	Up	1	Ethyl 1-aminocyclopropanecarboxylate	2.028	0.016	1.707
		Down	1	3-O-beta-D-galactosyl-sn-glycerol 2	2.996	0.000001	−1.727
			2	3,6-di-O-methyl-beta-D-glucose	2.913	0.00002	−2.929
			3	Propionic acid	2.478	0.004	−1.017
			4	Glutaral	2.507	0.001	−1.043
			5	Methylmalonic acid	2.480	0.004	−1.091
			6	N-methylethanolamine phosphate	2.234	0.006	−1.091
			7	Ethyl 1-aminocyclopropanecarboxylate	2.028	0.016	1.707
			8	L-BSO	2.112	0.020	−1.830
			9	O-(alpha-D-mannosyl)-L-threonine	1.983	0.022	−1.282
	12 dpi	Up	1	Pinolenic acid	2.393	0.019	1.423
			2	N-[5-(p-nitrobenzyloxy)-5-oxopentanoyl]glycine	2.252	0.030	1.132
		Down	1	3,6-di-O-methyl-beta-D-glucose	3.342	0.00000001	−3.946
			2	3-O-beta-D-galactosyl-sn-glycerol	3.326	0.00000001	−2.062
			3	Glycerol 3-phosphate	2.490	0.015	−2.254

*p*-value < 0.05; VIP, variable importance projection threshold: VIP score > 1.0; dpi, day post infection. Significant metabolites were filtered based on an absolute fold change (FC) ≥ 2.0 equivalent to |log_2_FC| > 1.0, representing a minimum two-fold difference in abundance. Positive values denote upregulation and negative values denote downregulation relative to the control group.
